# Implications of Metal Binding and Asparagine Deamidation for Amyloid Formation

**DOI:** 10.3390/ijms19082449

**Published:** 2018-08-19

**Authors:** Yutaka Sadakane, Masahiro Kawahara

**Affiliations:** 1 Graduate School of Pharmaceutical Sciences, Suzuka University of Medical Science, Suzuka 513-8670, Japan; sadapon@suzuka-u.ac.jp; 2 Department of Bio-Analytical Chemistry, Faculty of Pharmacy, Musashino University, 1-1-20 Shinmachi, Nishitokyo, Tokyo 202-8585, Japan

**Keywords:** Alzheimer’s disease, oligomerization, conformation, prion disease, iron

## Abstract

Increasing evidence suggests that amyloid formation, i.e., self-assembly of proteins and the resulting conformational changes, is linked with the pathogenesis of various neurodegenerative disorders such as Alzheimer’s disease, prion diseases, and Lewy body diseases. Among the factors that accelerate or inhibit oligomerization, we focus here on two non-genetic and common characteristics of many amyloidogenic proteins: metal binding and asparagine deamidation. Both reflect the aging process and occur in most amyloidogenic proteins. All of the amyloidogenic proteins, such as Alzheimer’s β-amyloid protein, prion protein, and α-synuclein, are metal-binding proteins and are involved in the regulation of metal homeostasis. It is widely accepted that these proteins are susceptible to non-enzymatic posttranslational modifications, and many asparagine residues of these proteins are deamidated. Moreover, these two factors can combine because asparagine residues can bind metals. We review the current understanding of these two common properties and their implications in the pathogenesis of these neurodegenerative diseases.

## 1. Introduction

Amyloids are fibril-like deposits observed in various tissues including the kidney, spleen, liver, and brain. In 1853, Virchow found abnormal accumulates in tissues and named them “amyloid”, since they exhibited similar characteristics to *amylum*, such as being stained by iodine [1]. In 1968, the major component of amyloid was determined to be proteins. The accumulation of amyloid in various organs causes disorders termed “amyloidosis”, including familial amyloid polyneuropathy (FAP), amyloid-light chain amyloidosis, and dialysis amyloidosis [2]. All of these diseases share common properties regarding the deposition of amyloids, which are protease-resistant, insoluble fibril-like structures (amyloid fibrils), and stained by congo-red (a β-sheet specific dye). Although the composition of amyloid is identical in each disease, some types of amyloidosis are fatal.

Recent neurochemical studies have suggested a link between amyloid formation and the pathogenesis of various neurodegenerative diseases such as Alzheimer’s disease (AD), prion diseases, Lewy body diseases (dementia with Lewy bodies (DLB)), etc. The disease-related proteins (amyloidogenic proteins), such as β-amyloid protein (AβP) in AD, prion protein in prion diseases, and α-synuclein in Lewy body diseases, share common characteristics, namely that they form amyloids with β-pleated sheet structures, and exhibit cytotoxicity in spite of possessing different primary sequences, as shown in Table 1. Thus, a new concept termed “conformational disease” was proposed, suggesting that protein conformation and its misfolding is an important determinant of its toxicity, and, consequently, the development of the related disease [3]. Considering that these amyloidogenic proteins are commonly found in our brain, factors that inhibit or accelerate the oligomerization process may play crucial roles in their neurotoxicity and pathogenesis [4]. As such, we focus here on two non-genetic factors: metal binding and deamidation of amino acid residues.

Considerable amounts of trace elements such as iron (Fe), zinc (Zn), copper (Cu), and manganese (Mn) exist in the brain, and the concentration and distribution of each metal differs in each brain region [5]. These essential trace elements play crucial roles in brain functions such as energy production, synthesis of neurotransmitters, and myelination. Because an excess or deficiency of these essential trace elements disrupts normal brain functions, their concentrations are strictly regulated.

**Figure 1:**
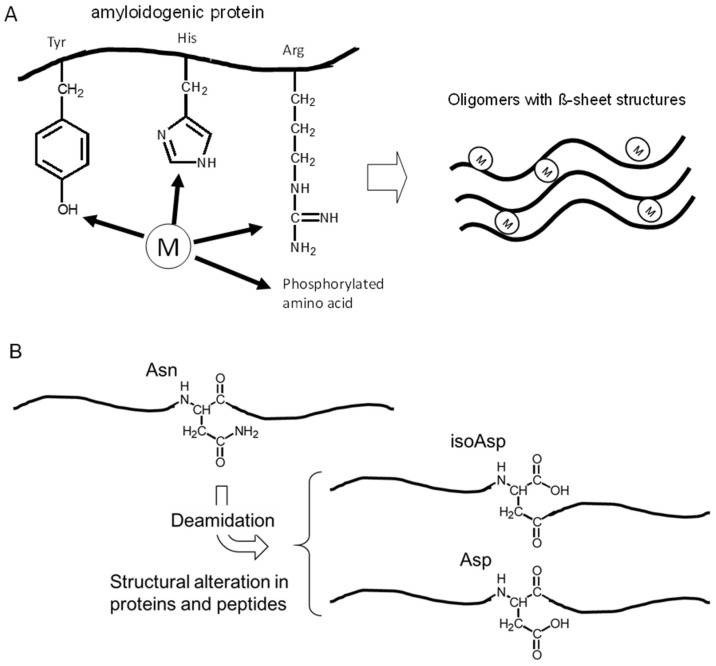
Metal binding and Asn deamidation in proteins. (**A**) Trace elements act as cross-linkers of amyloidogenic proteins. M stands for metal. (**B**) Deamidation of Asn residue affects the fibril formation by structural alteration of the neighboring Asn residue.

It is widely accepted that metal ions are essential factors for the regulation of protein conformations. These metals can firmly bind to metal-binding residues of proteins, such as arginine (Arg), tyrosine (Tyr), histidine (His), and phosphorylated amino acids residues, which can cause cross-linking of the proteins and influence their conformations (Figure 1A). Indeed, all of these amyloidogenic proteins possess the ability to bind metals, as shown in Table 1. Furthermore, most of these proteins, or the precursor proteins, are implicated in the maintenance of metal homeostasis [6]. We have observed metal-induced aggregation of amyloidogenic proteins using SDS-PAGE, thioflavin T (ThT) fluorescence assay, far-UV circular dichroism (CD) spectroscopy, and atomic force microscopy (AFM) imaging techniques [7,8,9,10].

Meanwhile, amyloidogenic proteins are susceptible to non-enzymatic posttranslational modifications caused by various stressors including oxidants, reducing sugars and reactive aldehydes owing to their long life span [11,12]. Asparagine (Asn) and aspartyl (Asp) residues are hotspots for such non-enzymatic posttranslational modifications, and the structural alterations of both residues are reported in many amyloidogenic proteins (Table 1). These alterations result in a change in local charge as well as the addition of extra carbon atoms to the polypeptide backbone, which is implicated in various biological phenomena including amyloid fibril formation (Figure 1B). For determining such structural alterations, we have developed a simple method using high-performance liquid chromatography (HPLC), which allowed us to analyze the amount of structurally-altered Asp residues in various proteins, including amyloidogenic peptide fragments [13,14].

In particular, the deamidation of Asn residues is a remarkable and prevalent phenomenon that occurs during protein aging, and has also been predicted by computer simulation. Over 170,000 Asn residues in 13,300 proteins for which the 3D structure is known were analyzed by an automated computational method. The calculated results revealed that at least one Asn residue in ~4% or ~17% proteins was estimated to undergo at least 10% deamidation within one or five days, respectively, under the physiological conditions [15]. More importantly, these predicted values accord quantitatively to the experimental results when the rates of over 1370 Asn deamidation are analyzed [16]. Taken together, considering the metal-binding ability of Asn residues, the interaction between metals and the deamidation may also occur in some diseases.

In this article, we review two common characteristics of amyloidogenic proteins, metal binding and Asn deamidation, and discuss their implications in the pathogenesis of conformational diseases.

## 2. Asparagine Deamidation and its Biological Significance

**Figure 2:**
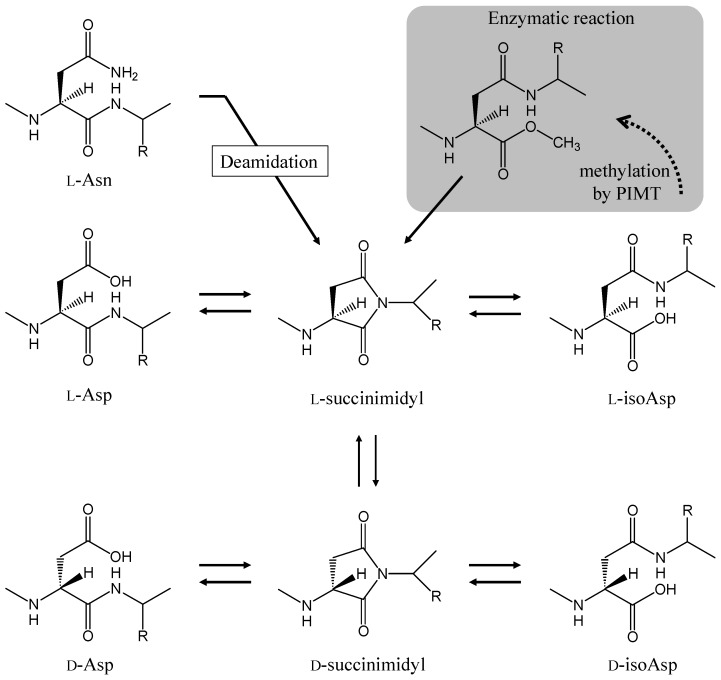
Pathways for spontaneous deamidation, isomerization, and racemization of l-Asn and l-Asp residues in the proteins. The PIMT repair system for l-isoAsp residue is also shown.

There are few reviews summarizing the relationship between amyloidogenesis and Asn deamidation. Therefore, we first introduce the mechanism that generates Asn deamidation and illustrate its biological significance with some examples. The deamidation of Asn residues occurs by intramolecular rearrangement, for example via a succinimide intermediate. The side chain carbonyl groups of the Asn residue is attacked by the peptide-bond nitrogen atom of the following residue, forming a five-membered succinimide ring intermediate [17] (Figure 2). The intermediate has a half-life of hours under physiological conditions before it is hydrolyzed, and a mixture of l-Asp and l-isoAsp is generated. The ratio of l-Asp to l-isoAsp is experimentally found to be approximately 3:1. Some l-succinimide intermediates also undergo reversible stereoinversion, which results in the formation of a d-succinimide intermediate. This intermediate is also quickly hydrolyzed, and a corresponding mixture of d-Asp and d-isoAsp is generated. Analyses using various model peptides reveal that the rate of succinimide formation is affected by both the primary amino acid sequence and the secondary structure of the protein. The amino acid residues on the carboxyl side of the asparagine/aspartyl (Asx) residue affect the succinimide formation rate. A fast rate is obtained when the carboxyl side residues are Gly, Ser, and Ala [18,19]. The succinimide formation is inhibited by higher order structures of protein such as α-helixes and β-sheets [13,20]. The half-lives of degradation of Asx residue vary between about 1 and 1000 days, depending on the circumstances of the Asx residue [21]. The succinimide formation rate of Asn residues is 10–30 times faster than those of Asp residue [18].

Since the deamidation of Asn is a dominant event for protein aging, the repair enzyme for isoAsp residue, which is the main primary reaction product of deamidation, is found in various organisms from bacteria to humans. Protein l-isoaspartyl *O*-methyltransferase (PIMT) catalyzes the transfer of a methyl group from *S*-adenosyl-l-methionine to the internal α-carboxyl group of a l-isoAsp residue (Figure 2). The methylation strongly accelerates the l-succinimide intermediate formation. The repetition of this repair cycle finally results in the replacement of an isoAsp with an Asp residue because the Asp residue is not a substrate for PIMT [22]. In knockout mice lacking PIMT, the amount of damaged protein containing the l-isoAsp residue is significantly increased in the brain, heart, liver, and erythrocytes in comparison to wild-type mice. The knockout mice show significant growth retardation and underwent several tonic-clonic seizures; they die at an average of 42 days after birth [23]. Proteomic approaches show that the substrates of PIMT are collapsin response mediator protein 2, dynamin 1, synapsin I, and synapsin II, which are characterized by having unique roles in neuronal function [24,25]. These show that deamidation of Asn residues is a critical event that causes protein functional disorder associated with various physiological systems, including neuronal dysfunction. The biological significance of deamidation will be explained in the following section with some examples.

The aggregation of eye lens crystallins, which may result in the formation of cataracts in aged lenses, is a specific example of a protein functional disorder caused by the deamidation of Asn residues. In human γS-crystallin, one of the major structural protein components of the eye lens, the deamidation of Asn^76^ causes a decrease in the stability of the protein and promotes dimer formation [26]. The deamidation of Asn^76^ and Asn^143^ enhances the protein-protein interaction, which leads to the promotion of protein aggregates [27]. αA-crystallin, obtained from elderly donors, contains d-Asp and d-isoAsp residues, and, interestingly, the d/l ratios of specific Asp residues are reported to be higher than 1.0 [28,29]. The dissociation of αA-crystallin is also significantly affected by the structural alteration of Asp residues [30]. The results obtained from two types of crystallins clearly show that structural alteration of Asx residues affects the function of the proteins [31]. Functional disorder, caused by Asn deamidation, is also reported in the case of calcium-modulated protein, calmodulin: specifically, the deamidation of two specific Asn residues caused a 90% reduction in activity of calmodulin [32]. The repair enzyme PIMT can partially recover the calmodulin activity (up to 40%) because PIMT promotes the conversion of l-isoAsp residues to l-Asp, but not to l-Asn. An examination using the *Xenopus* oocyte assay system also showed that the aged calmodulin became unstable, suggesting the alteration of calmodulin’s 3D structure by the deamidation of Asn residues [33]. On the contrary, it is reported that the deamidation of specific Asn residue causes protein function to be gained. Fibronectin is an adhesive protein that mediates various cellular interactions with the extracellular matrix. Although the Asn^263^-Gly-Arg (NGR) sequence of fibronectin is known to be crucial for the binding to the RGD-binding site of integrin, it is reported that the isoAsp-Gly-Arg (isoDGR) sequence, generated by Asn deamidation, is actually the sequence that binds to integrin [34]. In ceruloplasmin, a copper-binding protein with ferroxidase activity present in the cerebrospinal fluid (CSF), the ability to bind integrin also arises by the Asn deamidation of two internal NGR sites [35]. CSF obtained from AD patients promotes the deamidation of these two Asn residues because of its pathological pro-oxidative environment, suggesting that the environments of senile dementia promotes the integrin signaling pathway and cellular adhesion activity via deamidation of Asn residues.

## 3. Alzheimer’s Disease

### 3.1. Alzheimer’s Disease and β-Amyloid Protein

Senile type of dementia is an important problem for the elderly people worldwide. It is divided to AD, vascular type of dementia, and dementia with Lewy body, etc. AD shares the majority of senile dementia, and is characterized by the deposition of abnormal accumulated proteins, termed senile plaques and neurofibrillary tangles (NFTs) [36]. In AD brain, the loss of neurons and the degenerated synapses are also observed. NFTs are mainly constituted by phosphorylated tau proteins. The major component of senile plaques is AβP. Increasing evidence has suggested that the accumulated AβP and its neurotoxicity are based on the molecular pathogenesis of AD [37]. This idea, termed “amyloid cascade hypothesis”, has been reinforced by recent studies about the conformational changes of the identified AβP species [38].

**Figure 3:**
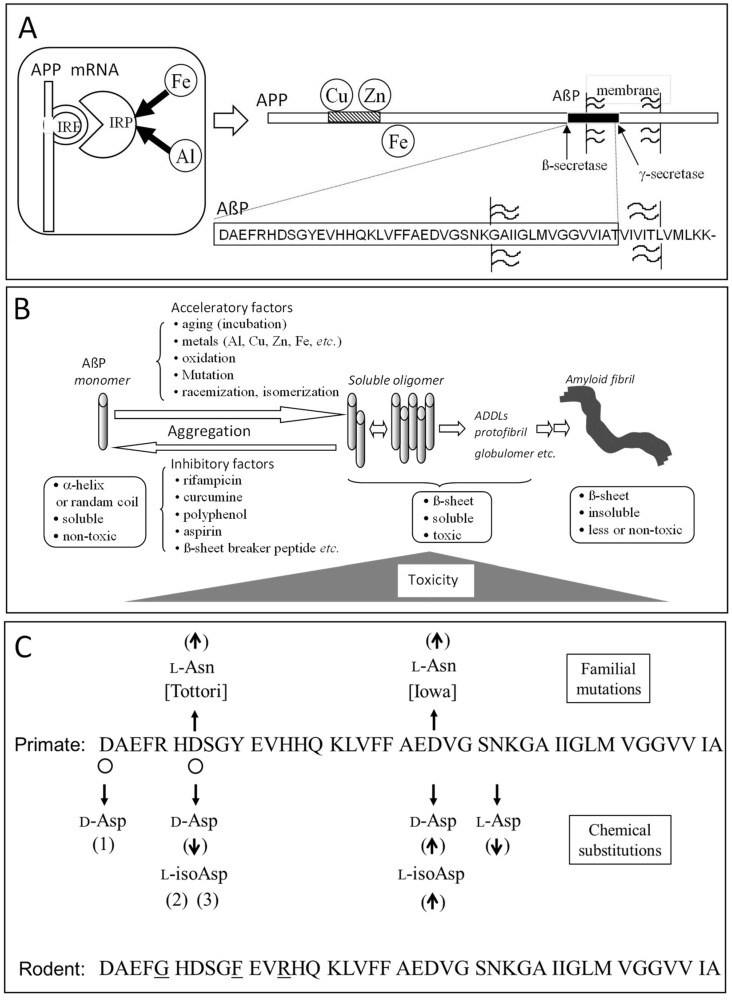
Alzheimer’s disease and factors affecting AβP aggregation. (**A**) Structure of APP and AβP. AβP is secreted by cleavage from its precursor protein, APP by transmembrane cleavage. The mRNA of APP possesses IRE domain, and Fe regulates its expression. (**B**) AβP aggregation. AβP self-aggregates and forms several types of oligomers (including SDS-soluble oligomers, ADDLS, globulomers, or protofibrils) and finally forms insoluble aggregates termed amyloid fibrils. Oligomeric soluble AβPs are toxic, although the monomeric and fibril AβPs are rather nontoxic. The aggregation process is influenced by the acceleratory factors or the inhibitory factors. (**C**) Summary of Asp isomerization in AβP. The Asp isomerization positions found in an AD brain are indicated by open circles. The relationship between chemical substitution and fibril formation is also shown. (↑) acceleration or increase of fibril formation, (↓) suppression or decrease, (1) suppression of acceleration effect by Asp^23^ substitution [41], (2) unchanged in vitro assay, (3) triggered the dense-core congophilic amyloid plaque formation in APP transgenic mice. The comparison between the sequence of primate (human or monkey) AβP^1–42^ and rodent (rat or mouse) AβP^1–42^ is also depicted and the different amino acids are indicated by underline.

As shown in Figure 3A, after the cleavage of N-terminus of amyloid precursor protein (APP) by β-secretase (BACE) and the intra-membrane cleavage of its C-terminal by γ-secretase, a 39–43 amino acid residue peptide (AβP) is secreted. It was reported that some familial AD patients possess APP mutations and the mutations in presenilin genes, which is a part of γ-secretase [39,40].

Neurotoxicity of AβP was first reported by Yankner et al. in 1991 [42]. However, there was a controversy about its neurotoxicity-aged AβP^1–40^, namely AβP^1–40^ incubated at 37 °C for several days was revealed to be more toxic to cultured neurons compared with freshly prepared AβP^1–40^ [43]. It was also demonstrated that β-sheet content of AβP, observed by CD spectroscopy, correlates with its neurotoxicity [44]. Based on these results, it became obvious that AβP easily form self-assembled aggregates (oligomers) with β-pleated sheet structures, and that the conformational change of AβP mainly contributes to its neurotoxicity. Furthermore, there are reportedly several types of soluble oligomer AβPs: naturally occurring soluble oligomers (dimers or trimers), AβP-derived diffusible ligands (ADDLs), AβP globulomers, and protofibrils [45]. Recent studies reinforced and modified the amyloid cascade hypothesis and exhibited the implication of AβP oligomers in the pathogenesis of AD (Figure 3B) [46].

### 3.2. Metals and AβP

AβP is reportedly secreted from APP into the brain of young people or of normal subjects [47]. Therefore, factors that accelerate or inhibit the aggregation process may become important determinants of the pathogenesis of AD. Various factors, such as mutations, oxidations of AβP, as well as environmental factors, such as pH, composition of solvents, concentrations of peptides, and temperature, all reportedly influence the aggregation processes (Figure 3B). Several small molecules, such as rifampicin, curcumin, carnosine, β-sheet breaker peptide, and aspirin, have been reported to inhibit AβP aggregation in vitro [48,49,50,51,52]. Some of these substances are considered to be protective agents against AD.

**Figure 4:**
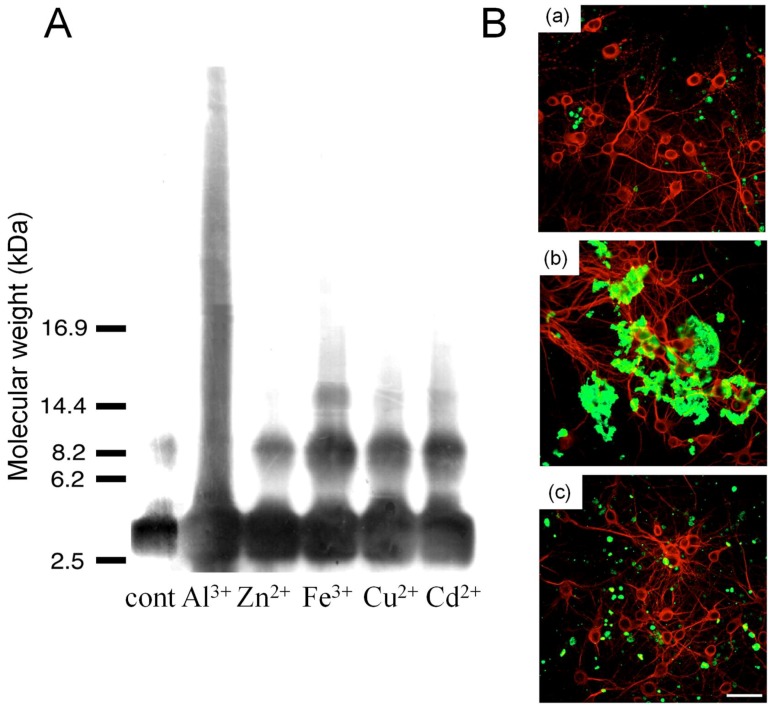
Metal-induced aggregation of AβP^1–40^. (**A**) Aggregation of AβP^1–40^ by various metals. The solutions of AβP^1–40^ were incubated at 37 °C for 24 h with or without various metal ions (each 1 mM), and were separated by SDS-PAGE using the tris-tricine method. (From [8], used with permission). (**B**) Deposition of AβP^1–40^ oligomers on neuronal membranes. The solutions of AβP^1–40^ were incubated at 37 °C for 24 h with Al^3+^ or Zn^2+^, and were applied onto cultured cortical neurons. After two days of exposure, cells were washed and double-immunostained with a polyclonal antibody to AβP (green) and a monoclonal antibody to MAP2 (red). The cells were observed under a confocal laser scanning microscopy. (**a**) Control, (**b**) Al-aggregated AβP, (**c**) Al-aggregated AβP. Bar represents 50 µm. (From [9], used with permission).

Among the factors that influence AβP aggregation, trace elements have been focused on for decades, since metals have the ability to crosslink the proteins and to cause their conformational changes, as shown in Figure 1A. Numerous studies reported the metal-induced aggregation of AβP. Exley et al. first demonstrated that Al^3+^ induces a conformational change in AβP^1–40^ by CD spectroscopy [53]. Furthermore, exposure to Al^3+^ causes the accumulation of AβP in cultured neurons or in the brains of experimental animals or humans. Alzheimer model mice transfected with the human APP gene (Tg 2576) exhibited a marked increase of secreted AβP as well as accumulated AβP and increased deposition of senile plaques after the administration of Al-contained foods [54]. We have investigated the metal-induced aggregation of AβP using SDS-PAGE and HPLC, and found that Al enhances the polymerization of AβP^1–40^ in vitro more than other metals, including Zn, Fe, Cu, and Cd [7,8,9] (Figure 4A). Aggregated AβP^1–40^ were heat- and SDS-stable; however, Al-aggregated AβP^1–40^ re-dissolves by deferoxamine, an Al chelator. Thus, it is possible that the binding between AβP aggregates is not covalent binding, but chelating. Moreover, Al-oligomerized AβPs exhibited fibrillar deposits on the surface of cultured neurons even several days after exposure (Figure 4B). Bush et al. found that Zn induced the aggregation of AβP, even at low concentrations (300 nM) [55]. They also reported that Cu markedly enhanced AβP aggregation [56]. Zn reportedly binds to three histidine residues (His^6^, His^13^, and His^14^) and/or to the carboxyl group of Asp^1^ of AβP [57]. Human AβP and rodent AβP are similar; however, AβP rarely accumulates in rodent (rat or mice) brains. Moreover, rodent AβP is less prone to aggregation compared with human AβP [58]. There are substitutions in three amino acid residues between human AβP and rodent AβP (Figure 3C). Interestingly, these three amino acids (Arg5, Tyr10, and His13) have the ability to bind metals (Figure 1A).

Moreover, the morphology of AβP aggregates treated with Al, Cu, Fe, and Zn was reported to be quite different [59]. The aggregation of AβP induced by trace metals including Al, Cu, Fe, and Zn and the neurotoxicity of the aggregated AβP are quite different [60]. Cu-aggregated AβP is reportedly more toxic than Zn-aggregated AβP [61]. Therefore, it is possible that these metals may be involved in the accumulation of human AβP. Indeed, the accumulation of these trace elements was observed in the senile plaques of AD patients [62,63].

Furthermore, APP also possesses the ability to bind metals, as shown in Figure 3A. APP reportedly possesses the ability to reduce Cu^2+^ to Cu^+^ [64]. Meanwhile, the expression of APP and the secretion of AβP are regulated by Zn and Cu [41]. It was also revealed that the trafficking of the APP from the endoplasmic reticulum to neurites is controlled by Cu [65]. APP binds to ferroportin, which controls Fe^2+^ efflux, and regulates Fe homeostasis [66]. Meanwhile, APP mRNA possesses an iron-responsive element (IRE) as well as ferritin (iron storage protein), and therefore, its expression is regulated by Fe [67]. These findings suggest that APP plays crucial roles in the regulation of metal homeostasis [6].

### 3.3. Isomerization and Racemization of Asp Residues in AβP

Delicate chemical analyses with AβPs isolated from the deposits reveal that the predominant component of aggregation is the 42 amino acid form of AβP (AβP^1–42^), and considerable structural rearrangements occur at Asp residues at the 1 and 7 positions of AβP, e.g., the Asp^7^ residue is changed to isoAsp (~70%) or Asp in the d-configuration (~10%) [68,69,70,71] (Figure 3C). The amount of isoAsp residue is considerably lower in the peptide isolated from cerebrovascular amyloid in comparison to that from parenchymal amyloid plaques [70]. Several familial AD (FAD) mutations are reported to occur within the AβP region of APP. The Tottori FAD is characterized by the intra-AβP missense mutation, causing the substitution of Asp^7^ for Asn (D7N), and the two affected sisters in the Tottori kindred show early-onset dementia, i.e., the age of onset is 60 and 65 years [72]. Both fibril formation and secondary structure transformation of the synthetic AβP (D7N) are accelerated in comparison to that of intact AβP [73,74]. The seeding effect on fibril formation also increases in AβP (D7N). Ion mobility mass spectrometry (IM-MS) reveals that the early aggregation state of AβP (D7N) is different from that of the intact peptide, while the monomer structures of both peptides have no difference [75]. Another FAD mutation causing the substitution of Asp^23^ for Asn (D23N), designated as Iowa, is also found, and characterized by showing symptoms of a progressive aphasic dementia, leukoencephalopathy, and occipital calcification [76]. Fibril formation of synthetic AβP (D23N) is remarkably accelerated and the protofibrils, which are observed as small globular oligomeric structures in the early stage of fibril formation, also appear earlier [77,78]. Both FAD mutations result in increased fibril formation, suggesting that the increment is one of the factors accounting for the early onset of AD.

The presence of the isoAsp residue is confirmed by PIMT assay in the tryptic AβP^17–28^ prepared from the brain of Iowa kindred [77], which is to be expected because the rate of succinimide formation is 10–30 times faster with an Asn residue than it is with an Asp residue. Analysis using a synthetic AβP (D7isoD and D23isoD) reveals that the isoAsp^23^ residue accelerates the fibril formation, but the isoAsp^7^ residue has little effect on the formation [79]. Immunohistochemical studies using anti-isoAsp antibodies showed that the senile plaques and vascular amyloid of an AD brain are stained by anti-isoAsp^23^ antibody, but those of a control brain are not. On the contrary, anti-isoAsp^7^ antibody stains the senile plaques and vascular amyloid of both AD and control brains [80]. In addition, AβP^1–42^ (D7isoD) acts as a trigger for the formation of dense-core amyloid plaques in the APP transgenic mouse brain [81]. The phosphorylation of proteins such as tau, tubulins, and matrin 3 is also accelerated by AβP^1–42^ (D7isoD) when the peptide is introduced to cultured cells [82]. The effect of Asp racemization on the fibril formation has also been examined using synthetic peptides. Stereoinversion of the Asp^23^ residue accelerates fibril formation, but that of Asp^7^ residue had little effect [83], and the stereoinversion of Asp^1^ residue strongly suppressed the acceleration of fibril formation by the d-Asp^23^ residue [84]. The drastically different rate change of fibril formation observed in AβP modified with isoAsp or d-Asp residue, as described above, suggests that the structural alteration of specific Asp residue may act as a potential trigger for AD amyloidosis. The fibril formation of AβP^1–40^ (N27D) is inhibited, indicating that the deamidation also affects the formation rate [85].

## 4. Prion Diseases

### 4.1. Pathogenesis of Prion Diseases and Prion Protein

**Figure 5:**
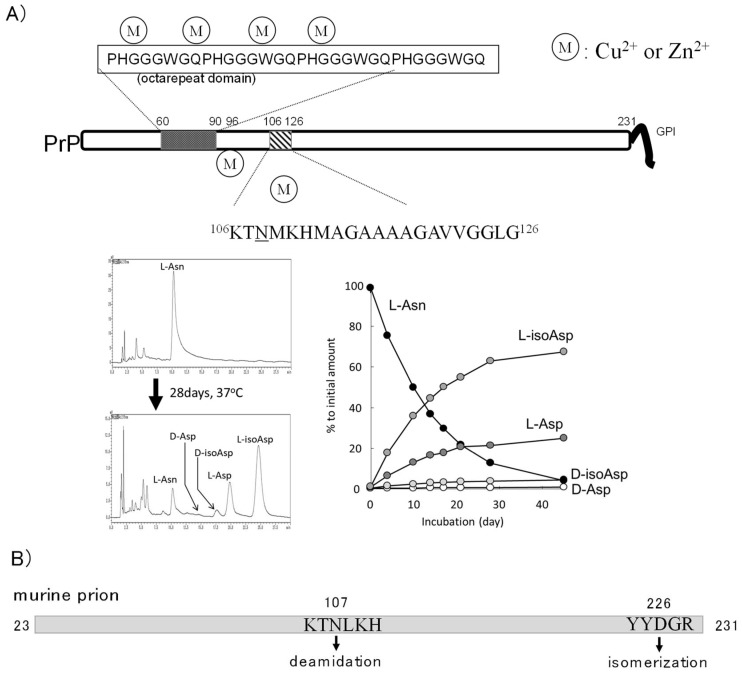
Prion protein structures and analysis of Asn deamidation. (**A**) The structure of PrP^C^ and metal-binding sites. Octarepeat domain and neurotoxic fragments PrP^106–126^ are depicted and Asn^108^ is indicated by underline. PrP^C^ possesses six metal-binding sites. The PrP^106–126^ was analyzed with a mobile phase containing 20% acetonitrile, a 15 mM sodium phosphate solution (pH 5.0), and 100 mM NaCl according to the methods described in [14]. The HPLC profiles after incubation for 28 days at 37 °C in 50 mM phosphate buffer (pH 7.4) and the summarized graph are shown (Y. Sadakane, unpublished data). (**B**) Asn deamidation and Asp isomerization in rodent prion protein are shown [87].

Prion diseases include scrapie (sheep), bovine spongiform encephalopathy (BSE, cattle), Creutzfeldt-Jakob disease (CJD, human), Gerstmann-Sträussler-Scheinker syndrome (GSS, human), and Kuru (human) [86]. Prion diseases are also called transmissible spongiform encephalopathies, since the spongiform degeneration of neurons and glia are commonly observed. Furthermore, in the patient brain, normal cellular prion protein (PrP^C^) converts to the abnormal scrapie-type isoform (PrP^Sc^) and accumulates. It is widely accepted that the invasion of of PrP^Sc^ from foods or from iatrogenic factors causes their infection characteristics. The misfolded and protease-resistant PrP^Sc^ promotes normal PrP^C^ in the brain to misfold and aggregate. PrP^C^ and PrP^Sc^ share the same chemical characteristics with the same primary sequence, except that PrP^Sc^ possesses high β-sheet contents. As shown in Figure 5A, PrP^C^ is a 30–35 kDa cell surface glycoprotein with a glycosylphosphatidylinositol (GPI) domain. Thus, it is widely accepted that the conformational change of PrP is a significant process in neurodegeneration and for the pathogenesis of prion diseases.

### 4.2. Prion Protein and Metals

There are several possible pathways in the neuro- or glia-toxicity of prion diseases. One possible pathway is the “loss of the normal, protective functions of PrP^C^”. Regarding this first pathway, increasing evidence suggests that normal PrP^C^ regulates metal homeostasis and possesses protective functions [88]. About the physiological roles of PrP^C^, Brown et al. reported that PrP-knockout mice exhibited the decreased Cu and the reduced activity of Cu-dependent enzymes [89]. They also demonstrated that PrP plays as a Cu/Zn superoxide dismutase (SOD) in the brain and has anti-oxidative stress roles [90]. PrP-deficient neurons are susceptible to free radicals such as hydrogen peroxide [91]. PrP^C^ regulates the excitability of *N*-methyl-d-aspartate (NMDA)-type glutamate receptor in a Cu-dependent manner [92]. Meanwhile, Cu^2+^ influences the gene expression and cellular trafficking of PrP [93]. Therefore, the depletion of PrP^C^ and the resulting Cu dyshomeostasis may trigger neurodegenerative processes.

As shown in Figure 5A, the octarepeat domain composed of multiple tandem copies of the eight-residue (PHGGGWGQ) of PrP^C^ reportedly binds 4 Cu in this octarepeat domain [94]. Other 2 His residues, His96, and His111 binds 2 Cu atoms. These binding sites can bind Zn^2+^, Mn^2+^, and Ni^2+^. The evolutionary similarities between prion genes and genes encoding Zrt-, Irt-like protein (ZIP)-type Zn transporters were demonstrated [95]. Watt et al. reported that PrP^C^ enhanced cellular uptake of Zn^2+^ via binding to the α-amino-3-hydroxy-5-methyl-4-isoxazolepropionate (AMPA)-type glutamate receptor, and that PrP^C^ acts as a Zn^2+^ sensor in the synapse [96]. Moreover, PrP reportedly facilitates Zn^2+^ influx into the brain and attenuates Zn-induced neurotoxicity. Therefore, PrP^C^ is also implicated in Zn homeostasis.

Furthermore, PrP^C^ reportedly has ferrireductase activity that converts Fe^3+^ to Fe^2+^, and then modulates the cellular uptake of Fe^2+^ [97]. Fe^2+^ ions are oxidized to Fe^3+^ by ferroxidases (such as ferritin or ceruloplasmin) in the bloodstream, then Fe^3+^ is transported with transferrin (an iron-binding protein that binds two Fe^3+^ ions) across the blood-brain barrier via transferrin receptors and enters neurons or glial cells. Then, Fe^3+^ is reduced to the bioactive Fe^2+^ by ferrireductase and transferred to neuronal enzymes, which require Fe^2+^ as a cofactor. Indeed, PrP-knockout mice exhibit altered Fe metabolism and Fe deficiency [98].

The second possible neurodegenerative pathway in prion diseases is a “gain of toxic functions of PrP^Sc^”. Metals are also implicated in the conformational changes and neurotoxicity of PrP^Sc^. Since whole PrP^Sc^ has strong infectious characteristics, synthetic fragment peptides of PrP, termed PrP^106–126^, are widely used as a model peptide of PrP^Sc^ to investigate PrP^Sc^ neurotoxicity [99]. PrP^106–126^ causes the apoptotic death of neurons or glial cells and possesses β-sheet structures [100]. PrP^106–126^ also possesses the ability to bind to metals, including Cu^2+^ and Zn^2+^ [101]. We have investigated the conformation of PrP^106–126^ and its neurotoxicity on primary cultured rat hippocampal neurons [10]. We have demonstrated that the co-existence of Zn^2+^ or Cu^2+^ significantly attenuated the neurotoxicity of PrP^106–126^ and that aggregation ability of PrP^106–126^ observed by ThT fluorescence. Furthermore, aged PrP^106–126^ in the presence of Cu^2+^ or Zn^2+^ exhibited different morphological features than aged PrP^106–126^ alone observed using AFM. Therefore, it is possible that Cu^2+^ and Zn^2+^ influenced the conformation and the neurotoxicity of PrP^106–126^.

### 4.3. Asn Deamidation in Prion Protein

Human or murine PrP possesses 11 or 13 Asn residues, respectively (Table 1). PIMT assay using the recombinant murine prion protein revealed that isoAsp residues increased the half-life of PrP to 33 days, and 0.8 mol isoAsp is accumulated per mol of protein after an incubation of 135 days [87]. The Asn^107^ residue is the main position for isoAsp accumulation, and Asp^226^, which exists only in murine PrP, is also isomerized. Asn^107^ residue is deamidated even in the PrP sample stored at −20 °C for several months; moreover, the deamidated Asn^107^ residue changes the sensitivity for metal ions [102]. Interestingly, the aged PrP, in which Asn^107^ is deamidated, forms aggregates and gains proteinase-K resistance in the presence of Cu^2+^. To quantify the structural alteration of Asp residues, we have established a simple method using reverse-phase HPLC, with a standard octadecylsilane column, and applied it to analyze the deamidation of Asn^108^ in human PrP^106–126^ peptide (or Asn^107^ in murine prion) [14]. Under physiological conditions, the Asn^108^ is deamidated with a half-life of 10 days (Figure 5A). The different half-lives between human and murine prions is due to the difference in the amino acid residues on the carboxyl side of the Asn residue and the highly ordered structure. d-Asp or d-isoAsp residues are also observed in PrP^106–126^ peptide after a 28-day incubation at 37 °C. Although the deamidated amino acids are accumulated with a comparatively high rate in Asn^108^ in human PrP, there is little information about the biological significance of deamidated PrP.

## 5. Lewy Body Diseases

**Figure 6:**
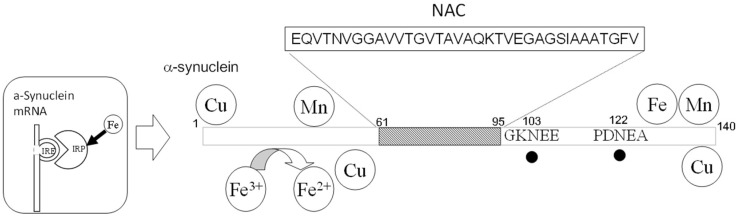
Structure of α-synuclein. The metal-binding sites and Asn deamidation sites are depicted. The sites of Asn deamidation are indicated by closed circles.

Lewy body diseases include Parkinson’s disease (PD), dementia with Lewy bodies (DLB), and multiple system atrophy, etc. [103]. Their common characteristic is the abnormal cellular inclusions called Lewy bodies, which are the accumulation of α-synuclein, and are therefore known as synucleinopathies. DLB accounts for approximately 25% of all senile dementia cases. Moreover, the α-synuclein fragment peptide non-amyloid component (NAC)-co-accumulates with AβP in the senile plaques of patients with AD. It is widely accepted that the aggregation of α-synuclein involves the molecular pathogenesis of Lewy body diseases, similar to AβP and PrP. α-Synuclein is abundant in the brain, primarily in presynaptic terminals, and is thought to play roles in maintaining the supply of synaptic vesicles to the presynaptic terminals and regulating the release of dopamine, as well as in synaptic functions and plasticity [104]. A comprehensive analysis with Fourier transform ion cyclotron resonance mass spectrometry (FT-ICR MS) reveals that two Asn residues at the 103 and 122 positions are deamidated dominantly with a half-life of 60 days in α-synuclein [105] (Figure 6). Furthermore, α-synuclein can bind metals including Cu, Mn, and Fe. α-Synuclein has ferrireductase activity that converts Fe^3+^ to Fe^2+^, and transfers bioavailable Fe^2+^ to many enzymes, such as thyrosine hydroxylase, and regulates the biosynthesis of neurotransmitters [106]. However, oligomerized α-synuclein reportedly has no ferrireductase activity [107]. As shown in Section 4.2, PrP^C^ has similar ferrireductase activity. Considering that α-synuclein is localized in the presynaptic domain and PrP^C^ is in the postsynaptic domain, these two amyloidogenic proteins may regulate neurotransmitter synthesis by controlling Fe^2+^/Fe^3+^ ratio in the synapse [6]. In contrast, Fe regulates the expression of α-synuclein because its mRNA has an IRE domain, similar to APP and ferritin [108].

## 6. Other Amyloidosis

**Figure 7:**
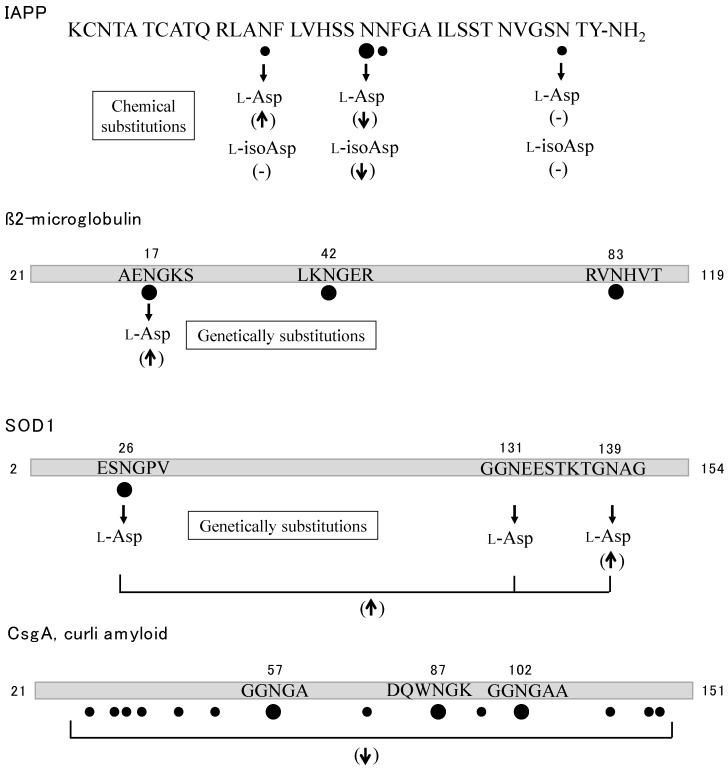
Summary of deamidation and isomerization in IAPP [112,113,114,115], β2-Microglobulin [116,117,118], SOD1 [119] and curli amyloid [120]. Asn deamidation and Asp isomerization are indicated by closed circles and open circles, respectively. The relationship between Asn substitution and fibril formation is also shown. (↑) acceleration or increase of fibril formation, (↓) suppression or decrease, (-) little or no effect.

Type 2 diabetes mellitus is characterized by the abnormal accumulation of islet amyloid polypeptide (IAPP or amylin) in the islets of Langerhans. IAPP is a 37-residue polypeptide with a disulfide bridge between the Cys^2^ and Cys^7^ residues, and acts as a partner hormone to insulin to control blood glucose concentration [109]. IAPP also has affinity for metals, such as Zn^2+^ or Cu^2+^ [110]. Cu^2+^ inhibits the formation of amyloid by IAPP and attenuates the cell toxicity of amylin [111]. IAPP incubated for several months under physiological conditions exhibits deamidation of at least four Asn residues at rates of 70–90%, and isotope-labeled IR spectroscopy shows that the fibril formation of deamidated IAPP was faster than that of intact IAPP [112] (Figure 7). Pramlintide is a synthetic analogue peptide of human IAPP in which residues 25, 28, and 29 are replaced with proline. To examine the stability, Asn deamidation was analyzed by HPLC-MS [113]. After incubation for 45 days at 40 °C, deamidation was found in four Asn residues, at positions 14, 21, 22, and 35 of pramlintide. The seeding effect was examined by using a short peptide fragment, IAPP^20–29^ [114]. While IAPP^20–29^ alone cannot form fibrils, a sample of peptide with as little as 5% deamidated peptide leads to the formation of amyloid deposits. The fibril formation of IAPP was examined by chemical substitution of Asn residues. IAPP peptides were successively prepared in which three Asn residues at the 14, 21, and 35 positions had been substituted with either l-Asp or l-isoAsp and then assayed by using ThT [115]. Substitution of N14D accelerates the fibril formation, the shape of which was long and thick; however, N14isoD shows little effect on fibril formation. Substitutions of N21D and N21isoD drastically inhibit the fibril formation; however, N35D and N35isoD show little effect.

β2-Microglobulin (β2M), which is a single-chain polypeptide composed of 99 amino acids, is a serum protein that serves as a component of major histocompatibility complex class I. Thus, β2M is required for antigen presentation in the immune responses. However, in some pathological conditions, the protein forms amyloid fibrils and is found as the major component of deposits associated with dialysis-related amyloidosis [121]. The heterogeneity of β2M is reported in patients treated with long-term hemodialysis. Several metals including Cu^2+^, Zn^2+^, Ni^2+^, and Al^3+^ can bind to β2M and influence the amyloid formation [122,123]. The sequencing analysis of the minor form of β2M reveals that it has identical amino acid sequence except for the substitution of Asn^17^ residue for Asp, suggesting that the Asn residue has been deamidated during long-term hemodialysis [116] (Figure 7). Asn deamidation of β2M was analyzed by a comprehensive top-down approach with FT-ICR MS [117]. Three Asn deamidation residues at the 17, 42, and 83 positions were found in aged β2M. The recombinant β2M (N17D) is able to form amyloid fibrils faster than the intact β2M under acidic conditions [118].

SOD1 is widely used in our bodies for protection against free radicals. It was the first identified protein for familial amyotrophic lateral sclerosis (ALS), which is a lethal neurodegenerative disease caused by the loss of motor neurons [124]. Over 180 different mutations in the *SOD1* gene have all been linked to ALS. The missense mutations such as N86D and N139D in SOD1, which cause familial forms of ALS, change the protein’s thermodynamic stability and folding behavior [125]. The deamidation rate of all seven Asn residues of SOD1 has been calculated by a computational method developed by Robinson et al. [126], which indicated that Asn residues at positions 26, 131, and 139 will be deamidated with 99%, 55%, and 21% conversion, respectively, after 450 days under physiological conditions. The lifetime of SOD1 is expected to be long enough to allow the accumulation of the deamidated residues. The recombinant protein (N139D) forms amyloid fibrils two times faster than that of intact SOD1, and the triple mutated protein (N26D, N131D and N139D) is also accelerated with an identical rate to that of N139D [119] (Figure 7). These results suggest that deamidation of Asn residues in intact SOD1 has the same effect on fibril formation as the familial SOD1 mutant (N139D).

Curli, observed in various bacteria including *Escherichia coli*, is a functional extracellular amyloid fiber and is associated with surface colonization and interaction with the host immune system [127]. The major curli subunit is CsgA protein, which has a self-polymerization ability, which allows it to form amyloid fibers. CsgA is composed of 130 amino acids and has 16 Asn residues. LC-MS/MS analysis reveals that deamidation is found in 14 Asn residues, and especially those at position 57, 87, and 102 are rapidly deamidated with a half-life of 2.5–5.3 days [120] (Figure 7). An aged CsgA incubated for 19 days shows little or no fibril formation, thus deamidation disables the fibril formation in bacterial curli.

## 7. Conclusions

Based on our findings and numerous other studies, amyloids share common characteristics such as metal binding and Asn deamidation. These two non-genetic factors can reflect the aging process, and influence the aggregation, conformation, and ultimately the neurotoxicity of amyloidogenic proteins. Moreover, as we have shown here, metal-binding Asn residues can be deamidated, and the combination of these two factors profoundly affects amyloidogenesis, such as the effect of Cu^2+^ on the aged murine PrP [102]. The deamidation of Asn residues is a reaction that is mediated by water and hydroxide ions, but other post-translational chemical modifications require specific factors such as reactive oxygen species, lipids, and enzymes. Thus, Asn deamidation occurs throughout the life time of a protein. The calculated results reveal that Asn deamidation progresses to at least a 10% level in 1/20 of total proteins within one day under physiological conditions [15]. This shows that, even early on, a small amount of deamidated product may be present in vivo. It is widely accepted that a small portion of aggregation can act as a seed to propagate the amyloid fibril formation. The deamidated product, which often shows rapid fibril formation, may be one of the main actors for the seeded propagation contributing to the progression of conformational diseases. In actuality, low levels of deamidation impurities (less than 5%) of the IAPP fragment peptide are reported to be able to accelerate fibril formation [114].

Two FAD mutations, Tottori and Iowa, are reported to be present in early-onset dementia and to cause the substitution of Asp^7^ or Asp^23^, respectively, for the Asn residue in the AβP region of APP [72,73,74,75,76,77,78]. The Asn residues show very fast succinimide formation comparing Asp residues [18]; consequently, isoAsp residues are rapidly accumulated in AβP. The synthetic AβPs in which Asp^7^ or Asp^23^ is substituted for isoAsp residue show the enhancement of neurotoxicity [82] or the acceleration of fibril formation [83], respectively. These results suggest that both FAD mutations in AβP accelerate amyloid fibril formation in the brain of patients owing to the fast generation of the isoAsp residue in the AβP of Tottori and Iowa. However, whether the structural alteration of Asx resides in amyloidogenic proteins as a cause or a consequence of amyloid fibril formation remains to be fully clarified. Further research is needed to obtain direct in vivo evidence that structural alteration of Asx resides including Asn deamidation acts as a seed for amyloid fibril formation.

Considering two common characteristics, we hypothesized that the pathogenesis of conformational diseases is linked to metals and deamidation. This hypothesis may help in the development of drugs for these diseases, so that substances that influence these two factors may become candidates for the treatment of these diseases. We have already focused on carnosine as one such substance because it possesses various beneficial attributes such as metal-chelating, antioxidant, anti-glycation, and anti-crosslinking [128,129]. In particular, carnosine is used for the treatment of cataracts owing to its anti-crosslinking ability [130]. The protective activity of carnosine against the accumulation of AβP has also been reported [50]. Concerning substances that can suppress Asn deamidation, further research is urgently required because there are few reports in this under-researched field. Further research about the detailed characteristics, including the conformation and the toxicity, of deamidated amyloids is necessary.

## Figures and Tables

**Table 1:** Characteristics of amyloidogenic proteins and related peptides.

Disease Name Sequence	Binding Metals Structural Alteration of Asn or Asp	Functions of Amyloidogenic Proteins or Their Precursors
**Alzheimer’s Disease** AβP^1–42^	Al, Zn, Cu, Fe Isomerization and racemization of Asp^1^ and Asp^7^	✓ Neuronal proliferation and development ✓ Neurite outgrowth ✓ Fe homeostasis
***D***AEFRH***D***SGYEVHHQKLVFFAEDVGSNKGAIIGLMVGGVVIA		
**Prion Diseases** Prion protein; (PrP^106–126^)	Zn, Cu, Fe, Mn Deamidation of Asn^108^	✓ SOD activity ✓ Cu homeostasis ✓ Zn homeostasis ✓ Fe homeostasis and ferrireductase activity
KKRPKPGGWNTGGSRYPGQGSPGGNRYPPQGGGGWGQPHGGGWGQPHGGGWGQPHGGGWGQPHGGGWGQGGGTHSQWNKPSKPKT***N***MKHMAGAAAAGAVVGGLGGYMLGSAMSRPIIHFGSDYEDRYYRENMHRYPNQVYYRPMDEYSNQNNFVHDCVNITIKQHTVTTTTKGENFTETDVKMMERVVEQMCITQYERESQAYYQRGS		
**Lewy Body Diseases** α-synuclein; (*NAC*, a *fragment of* α-*synuclein*)	Cu, Fe, Al Deamidation of Asn^103^ and Asn^122^	✓ dopamine release ✓ Fe homeostasis and ferrireductase activity
MDVFMKGLSKAKEGVVAAAEKTKQGVAEAAGKTKEGVLYVGSKTKEGVVHGVTTVAEKTKEQVSNVGGAVVTGVTAVAHKTVEGAGNFAAATGLVKKDQK***N***ESGFGPEGTME***N***SENMPVNPNNETYEMPPEEEYQDYDPEA		
**Type 2 Diabetes** Islet amyloid peptide (IAPP, amylin)	Cu, Zn Deamidation of Asn^21^	✓ a partner hormone to insulin to control blood glucose concentration
KCNTATCATQRLANFLVHSS***N***NFGAILSSTNVGSNTY		
**Dialysis Amyloidosis** β2-microglobulin	Al, Cu, Zn, Ni Deamidation of Asn^17^, Asn^42^ and Asn^83^	✓ antigen presentation in the immune responses
IQRTPKIQVYSRHPAE***N***GKSNFLNCYVSGFHPSDIEVDLLK***N***GERIEKVEHSDLSFSKDWSFYLLYYTEFTPTEKDEYACRV***N***HVTLSQPKIVKWDRDM		
**Amyotropic Lateral Disorder (ALS)** Cu, Zn-SOD1	Cu, Zn Deamidation of Asn^26^	✓ SOD activity ✓ cellular defense
ATKAVCVLKGDGPVQGIINFEQKES***N***GPVKVWGSIKGLTEGLHGFHVHEFGDNTAGCTSAGPHFNPLSRKHGGPKDEERHVGDLGNVTADKDGVADVSIEDSVISLSGDHCIIGRTLVVHEKADDLGKGGNEESTKTGNAGSRLACGVIGIAQ		

The sequence of fragment peptide of each amyloidogenic protein (PrP^106–126^, NAC) is indicated by an underline. Deamidated Asn or Asp residues are shown as italic bold. In “Functions” of Alzheimer’s disease, possible functions of APP are noted. The numbers in the upper right indicate residues’ range. Same as below.

## Abbreviations

AβP	β-amyloid protein
AD	Alzheimer’s disease
AFM	Atomic force microscopy
ALS	Amyotrophic lateral sclerosis
AMPA	α-amino-3-hydroxy-5-methyl-4-isoxazolepropionate
APP	Amyloid precursor protein
β2M	β2-Microglobulin
BSE	Bovine spongiform encephalopathy
CD	Far-UV circular dichroism
CJD	Creutzfeldt-Jakob disease
CSF	Cerebrospinal fluid
DLB	Dementia with Lewy bodies
FAP	Familial amyloid polyneuropathy
FT-ICR MS	Fourier transform ion cyclotron resonance mass spectrometry
GPI	Glycosylphosphatidylinositol
GSS	Sträussler-Scheinker syndrome
HPLC	High-performance liquid chromatography
IAPP	Islet amyloid polypeptide
IM-MS	Ion mobility mass spectrometry
IRE	Iron-responsive element
NAC	Non-amyloid component
NFT	Neurofibrillary tangles
NMDA	*N*-methyl-d-aspartate
PD	Parkinson’s disease
PIMT	Protein l-isoaspartyl *O*-methyltransferase
PrP	Prion protein
SOD	Superoxide dismutase
ZIP	Zrt-, Irt-like protein
ThT	Thioflavin T
